# The First Report of Enterobacter Endosymbionts in the Dried Fruit Mite (*Carpoglyphus lactis* L.) (Acari, Acarida) Reared on Apricots in the Laboratory

**DOI:** 10.1111/1758-2229.70294

**Published:** 2026-02-18

**Authors:** Majid Rakhshandeh, Mohammad Khanjani

**Affiliations:** ^1^ Department of Plant Protection Faculty of Agriculture, Bu‐Ali Sina University Hamedan Iran

**Keywords:** 16S rRNA, allergenicity, *Carpoglyphus lactis*, *Enterobacter hormaechei*, *gapA*, microbiome, storage mites

## Abstract

*Carpoglyphus lactis* (Linnaeus), a member of the family *Carpoglyphidae*, is recognised both as a common storage mite and a significant source of indoor allergens. Despite extensive studies on its biology and distribution, little is known about its associated microbiome. In this study, for the first time, we investigated the bacterial symbionts of *C. lactis* reared under sterile laboratory conditions on dried apricots. Following surface sterilisation, bacterial isolates were cultured and identified through biochemical tests and molecular analyses targeting the 16S rRNA and *gapA* genes. Phylogenetic analyses revealed that the isolated strains shared over 98% similarity with *Enterobacter hormaechei* and clustered specifically within the *E. hormaechei* subsp. *xiangfangensis* clade. These findings confirm the presence of *Enterobacter* species as endosymbionts in *C. lactis* for the first time. The symbiotic relationship may contribute to host stress tolerance, nutritional efficiency and modulation of allergenic properties. This discovery opens new avenues for exploring mite–microbe interactions and developing innovative strategies for biological control and allergy mitigation.

## Graphical abstract

This study reports for the first time the presence of *Enterobacter hormaechei subsp. xiangfangensis* as an endosymbiont in the dried fruit mite, *Carpoglyphus lactis*, reared on apricots under sterile laboratory conditions. Through surface sterilisation, bacterial culturing and molecular identification (16S rRNA and gapA gene analysis), the bacterial strains were confirmed and phylogenetically classified. The identified symbiosis suggests potential roles in stress tolerance, improved nutrition and allergen modulation in *C. lactis* . These findings offer new insights into mite–microbe interactions and their possible implications in allergy control and biological strategies.

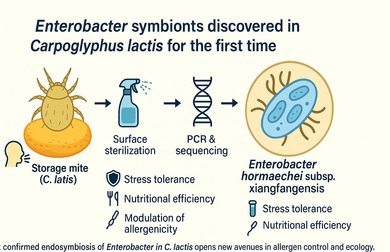

## 1 Introduction

Mites (Acari) represent one of the most diverse groups within the arthropods, occupying a wide range of terrestrial and aquatic habitats and contributing significantly to global biodiversity (Xie and Zhang 2023; Ozman‐Sullivan and Sullivan 2024). It is estimated that over 55,000 species have been described to date, while many more remain unidentified (Dhooria 2016). These organisms exhibit remarkable ecological adaptability, allowing them to thrive not only in soil and water but also as ectoparasites on plants and animals (Dowling 2015). Among those coexisting with humans, allergenic mites have gained particular attention due to their considerable impact on public health. They are recognised as major sources of household allergens, playing a crucial role in the development of allergic respiratory diseases such as allergic rhinitis and asthma (Pawankar et al. 2009; Miller 2019; Colloff 2009; Zock et al. 2002; Acevedo et al. 2019; Yu et al. 2014). Warm and humid conditions, the presence of organic food sources and enclosed environments such as homes and dormitories provide ideal habitats for these mites (Wright et al. 2009; Wilson and Platts‐Mills 2018; Ricci et al. 2001; Fukutomi and Kawakami 2021).

The order Astigmata, particularly families associated with humans, is recognised as the most prevalent group in urban environments (Hubert et al. 2012; O’Connor 2009; Solarz et al. 2004). Among these, the family Carpoglyphidae (Oudemans 1923), which comprises four main genera—*Carpoglyphus* (Robin 1868), *Dichotomiopus* (Fain and Camerik 1978), *Pullea* (Canestrini 1884) and *Coproglyphus* (Türk and Türk in Stammer 1957)—has garnered significant attention due to its role in the degradation of stored food and the induction of respiratory allergies (Aliakbarpour and Fan 2022; A. M. Hughes 1961; Bell 2011). Mites belonging to this family, especially *Carpoglyphus lactis* (L.), are of particular importance in public health and food safety, owing to their dual role as both stored‐product pests and sources of domestic allergens (Aliakbarpour and Fan 2022; Hubert et al. 2015). *C. lactis* feeds on dry food materials such as dates, grains, powdered milk and dried fruits, and is also found in household dust, contributing to its widespread distribution (Halliday 2003; Taha et al. 2019). Its high adaptability to warm and humid conditions facilitates dense population buildups in various settings, which may in turn intensify symptoms of allergic diseases such as rhinitis, asthma and atopic dermatitis (Colloff 2009; Acevedo et al. 2019; Yu et al. 2014).

Despite previous studies on the ecology, morphology and distribution patterns of *Carpoglyphus lactis* (Hubert et al. 2015; Saha 2016), limited information is available regarding its associated microbiome composition. Increasing evidence suggests that symbiotic microbiota play crucial roles in the biology, physiology, environmental stress tolerance and even allergenic properties of mites (Yan et al. 2025; Andrianov 2022). While the microbiome's influence on biological traits has been well documented in other mite species, such as *Phytoseiulus persimilis* (Yan et al. 2025), such data remain scarce for *C. lactis* . Morphologically, *C. lactis* is a small mite, measuring approximately 200–300 μm in body length, with a colour ranging from yellowish‐white to light brown. Its life cycle comprises four stages: egg, larva, nymph and adult, which can be completed within 2–4 weeks under favourable environmental conditions (Bakr et al. 2021; Chmielewski 1999; Abu‐EINour 2005; Hubert et al. 2015, 2018, 2021; Kucerova and Stejskal 2009; Zhang et al. 2025).

Recently, increasing attention has been directed toward the role of the mite‐associated microbiome and its influence on mite biology and physiological traits. Numerous studies have demonstrated that symbiotic microorganisms can impact mite nutrition, development, thermal tolerance and resistance to pathogens (Yan et al. 2025; Andrianov 2022). For instance, Yan et al. (2025) reported the presence of *Stenotrophomonas maltophilia* in the predatory mite *Phytoseiulus persimilis*, highlighting its contribution to enhanced stress resistance in the host. Regarding *Carpoglyphus lactis*, Hubert et al. (2015) examined the mite's microbiome in dried fruits and found that diet composition significantly influenced the diversity and structure of the associated bacterial communities. Identified bacteria included members of the genera *Bacillus*, *Kocuria*, *Erwinia*, *Serratia*, *Pseudomonas*, *Enterococcus*, *Rhizobium* and *Streptococcus*, among others. However, to date, no reports have confirmed the presence of *Enterobacter* species in association with *C. lactis* .

Given the growing recognition of the microbiome's role in regulating the biology of allergenic mites, identifying and analysing the microbial diversity associated with *Carpoglyphus lactis* can offer deeper insight into the mechanisms of allergenicity and support the development of effective control strategies. Accurate characterisation of the mite‐associated microbiome may provide a powerful tool for understanding the biological functions, survival and allergenic potential of mites. However, a significant gap remains in our current understanding of the microbial diversity associated with *C. lactis* . In particular, the potential role of microorganisms such as *Enterobacter* spp. in influencing the physiological traits of this species has yet to be explored. Therefore, the present study was conducted to identify and characterise the symbiotic microbiome of *C. lactis* . For the first time, this investigation reports the isolation and identification of *Enterobacter* spp. from *C. lactis* , which may serve as a basis for future studies on mite–microbe interactions and innovative approaches to biological control and allergy mitigation.

Bacteria of the genus *Enterobacter*, belonging to the *Enterobacteriaceae* family, are frequently found in the microbiomes of invertebrates, particularly mites and play significant roles in the ecology and physiology of their hosts. These Gram‐negative, rod‐shaped, facultatively anaerobic bacteria are widespread in environments such as soil, water and sewage, and are also natural inhabitants of the gastrointestinal tract of humans and animals (Davies and Davies 2010; Sanders Jr and Sanders 1997).

Clinically, *Enterobacter* species are recognised as opportunistic pathogens capable of causing a wide range of nosocomial infections, including urinary tract infections, wound infections, sepsis and pneumonia (Mezzatesta et al. 2012). Particularly concerning are carbapenem‐resistant *Enterobacteriaceae* (CRE), which pose a serious threat to public health due to limited treatment options (Nordmann et al. 2012).

## 2 Materials and Methods

### 2.1 Mite Rearing

*Carpoglyphus lactis* specimens were obtained from the Hekmataneh Biological Control Agents Company and reared under sterile conditions in food containers containing a mixture of flour, yeast, dried apricot and bran. The mites were maintained in an incubator at controlled environmental conditions of 25°C ± 2°C temperature and 85% ± 2% relative humidity, in complete darkness (reflecting their stored‐product habitat). To eliminate the potential effects of the original food substrate, the mites were reared for five consecutive generations. Furthermore, to ensure the removal of prior dietary influences, mite eggs were transferred to a fresh rearing container, and only individuals that hatched from these eggs were used for colony maintenance and subsequent experimental assays. This procedure ensured dietary standardization across experimental individuals.

### 2.2 Surface Sterilisation of Mites

#### 2.2.1 Sterilisation With 70% Ethanol

Approximately 100 adult *C. lactis* mites were gently collected from the rearing medium using a fine sterile brush and transferred into a sterile beaker containing sterile distilled water to remove surface debris and rearing residues. The mites were then carefully transferred into a 1.5 mL sterile microtube containing distilled water. To further eliminate impurities, the sample was centrifuged at 5000 rpm for 60 s. After gently discarding the supernatant, 1000 μL of 70% ethanol was added to the microtube, and the contents were incubated for 60 s, followed by another round of centrifugation at 5000 rpm. The ethanol was discarded, and the mites were rinsed three times with sterile distilled water under the same centrifugation conditions. Finally, the microtube was opened and placed under a laminar flow hood at room temperature to allow complete drying of the mites.

#### 2.2.2 Sterilisation With 2% Sodium Hypochlorite

This procedure was performed in the same manner as ethanol sterilisation, with the substitution of 70% ethanol by 2% sodium hypochlorite for the disinfection step.

### 2.3 Bacterial Culturing

Surface‐sterilised mites were transferred onto Nutrient Agar (NA) plates using a sterile needle. The plates were then incubated at 25°C for 24 h. After incubation, visible bacterial colonies were examined. For purification, representative colonies were subcultured onto fresh NA plates. Streak plating (lawn culture technique) was used to isolate and purify single bacterial strains.

Two negative controls were included in the experiment: (1) NA plates without mites (blank medium control), and (2) NA plates inoculated with sterile water obtained from the mite washing process. Both controls were used to confirm that no bacterial contamination occurred during the handling and sterilisation procedures.

### 2.4 Molecular Identification

#### 2.4.1 Genomic DNA Extraction From Bacterial Strains

Genomic DNA was extracted from bacterial strains using the alkaline lysis method described by Ausubel et al. (1992). Initially, bacterial strains were cultured on NA medium. After 10 days of incubation and colony growth, a small piece of the bacterial colony was transferred into a microtube. Then, 1000 μL of TE buffer was added to the tube, which was subsequently boiled in a water bath for 5 min and immediately cooled on ice. This boiling and cooling cycle was repeated three times consecutively. The extracted genomic DNA was then used for Polymerase Chain Reaction (PCR) assays.

#### 2.4.2 Assessment of Extracted DNA Quality

To determine the quantity and quality of the extracted DNA, the absorbance of the samples was measured at wavelengths of 260 nm and 280 nm using a Nanodrop spectrophotometer. Samples with an A260/A280 ratio between 1.8 and 2.0 were considered to have high‐quality DNA suitable for downstream applications.

#### 2.4.3 PCR Reaction and Optimisation Conditions

Partial regions of the 16S rRNA and gapA genes were amplified using primers 27F and 1492R for 16S rRNA, and gapA326F and gapA845R for gapA, respectively (Heuer and Smalla 1997) (Table 2). The PCR reaction mixture was prepared using a ready‐to‐use PCR master mix (Ampliqon, Denmark) with a total volume of 25 μL, containing 12.5 μL of master mix, 1 μL of genomic DNA at a concentration of 300 ng/μL, and 1 μL of each primer at a concentration of 10 pmol. The final volume was adjusted with deionised water. Amplification was performed in a thermal cycler (TECHNE, England) under the following conditions: initial denaturation at 95°C for 3 min, followed by 30 cycles of denaturation at 95°C for 30 s, annealing at 58°C for 16S rRNA and 61°C for gapA primers for 30 s, and extension at 72°C for 60 s, with a final extension step at 72°C for 5 min.

#### 2.4.4 Electrophoresis of PCR Products and Sequencing

To visualise the PCR products, 3 μL of each PCR reaction mixture was loaded into wells of a 1% agarose gel prepared with TAE buffer. Electrophoresis was conducted for 1.5 h at a constant voltage of 80 V. Following electrophoresis, the gel was stained with 0.5 μg/mL ethidium bromide solution to visualise the amplified DNA bands. Images of the gel were captured using a gel documentation system (Ausubel et al. 1992). The PCR products were subsequently sent to Bioneer Company (South Korea) for Sanger sequencing.

### 2.5 Phylogenetic Tree Construction

**FIGURE 1:**
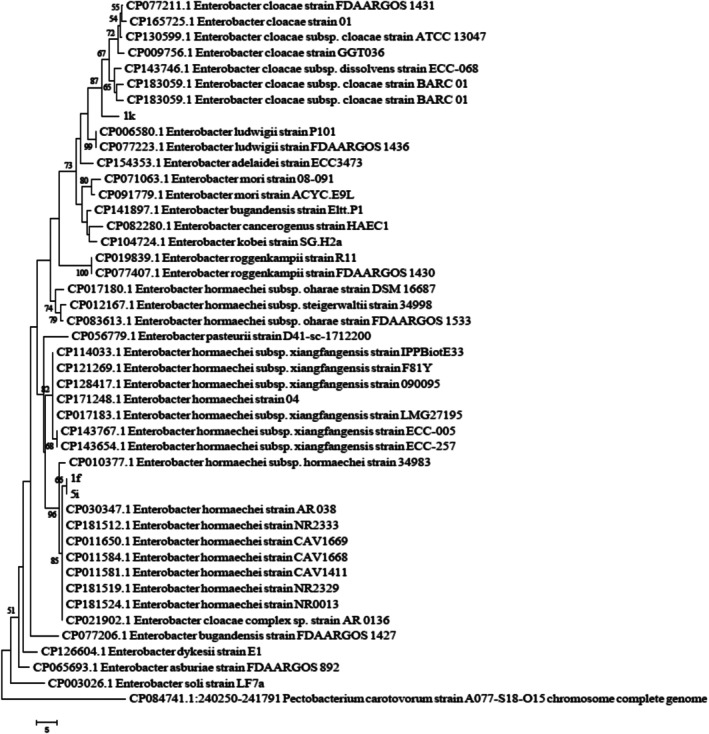
The phylogenetic tree of strains 1f, 1k and 5i was constructed using the neighbour‐joining method based on the 16S rDNA gene. The scale bar represents 0.01 nucleotide substitutions per site between different strains. Numbers above the branches indicate bootstrap support values based on 1000 replicates.

**FIGURE 2:**
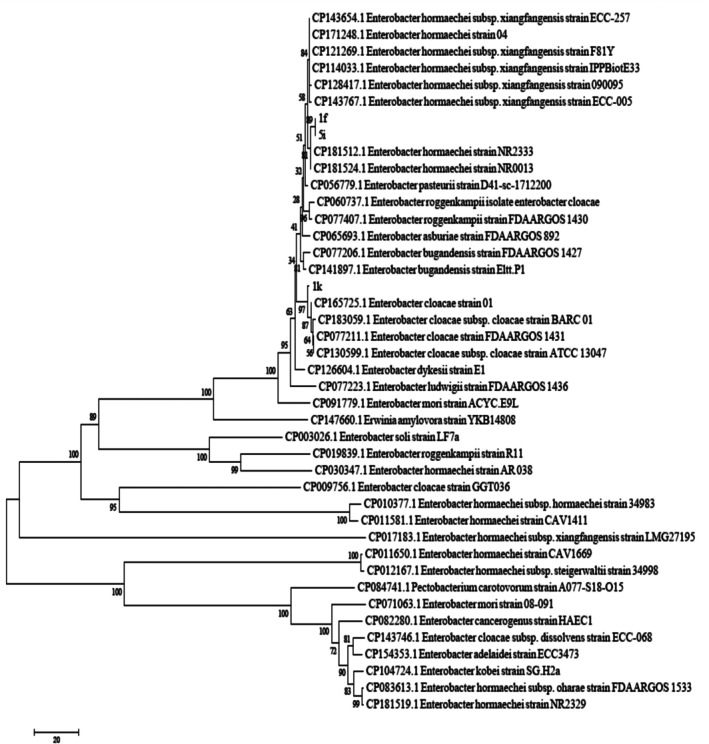
Phylogenetic tree of strains 1f, 1k and 5i constructed using the neighbour‐joining method based on the *gapA* gene. The scale bar represents 0.01 nucleotide substitutions per site among different strains. Bootstrap values above each branch indicate support levels based on 1000 replicates.

**FIGURE 3:**
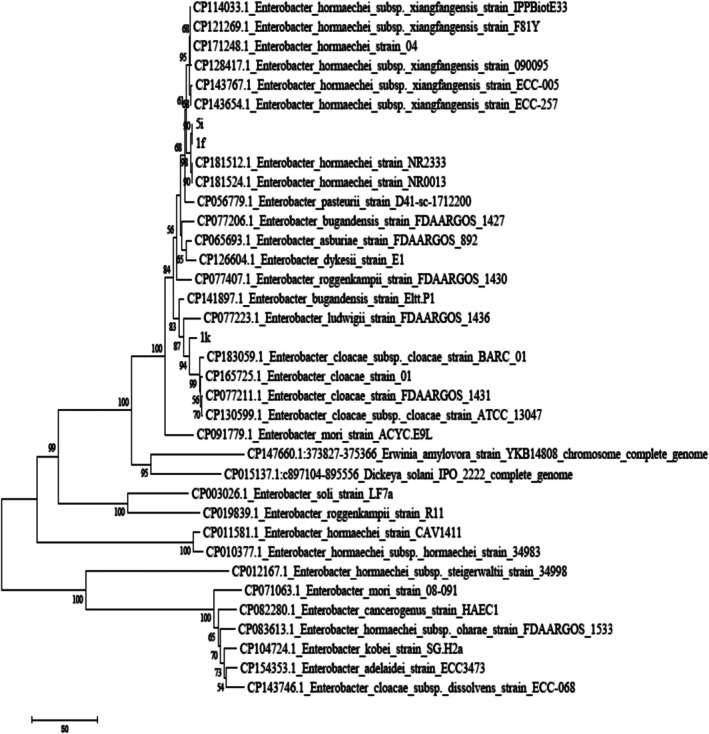
Phylogenetic tree of strains 1f, 1k and 5i constructed using the neighbour‐joining method based on the combined sequences of the *16S rDNA* and *gapA* genes. The scale bar represents 0.01 nucleotide substitutions per site among different strains. Bootstrap values shown above the branches represent support based on 1000 replicates.

The nucleotide sequences obtained from the examined isolates were edited using BioEdit Sequence Alignment Editor software (version 7.0.5.3) and submitted to the GenBank database. To compare sequence similarity with reference strains, the BLASTn tool in the NCBI database was utilised Phylogenetic analyses were performed using MEGA software (version 6). Initially, sequences were aligned with the ClustalW algorithm and manually inspected. The best nucleotide substitution model was selected based on the Bayesian Information Criterion (BIC). Subsequently, the phylogenetic tree was reconstructed using the Subtree‐Pruning‐Regrafting (SPR) method with 1000 bootstrap replicates. For rooting the tree, *Pectobacterium brasiliense* strain PCC1 from the Pectobacteriaceae family was chosen as the outgroup (Figures 1, 2, 3).

### 2.6 Antibiotic Susceptibility Test

To verify the endosymbiotic nature of *Enterobacter hormaechei* in the mite *C. lactis* , the antibiotic susceptibility of the bacterium was tested against gentamicin (GM), tetracycline (TE) and streptomycin (S), with a control group lacking antibiotic exposure. Each treatment included five biological and two optional technical replicates.

Bacterial growth inhibition was assessed using the disk diffusion method on NA plates inoculated with *E. hormaechei* . The inhibition zone diameters were measured after incubation, and larger zones indicated higher antibacterial activity. Data were analysed by one‐way ANOVA followed by Tukey's post hoc test (Montgomery 2017). The antibiotic with the strongest inhibitory effect was selected for subsequent experiments.

### 2.7 Feeding Assay on Mites

In the second stage, the role of *E. hormaechei* as an endosymbiont of *C. lactis* was examined through a feeding assay. Two groups of mites were maintained under identical conditions (25°C ± 2°C and 70% relative humidity):

1. *Control group*: fed with a diet lacking antibiotics.
2. *Treatment group*: fed with a diet containing streptomycin.

After 48 h, mites from both groups were collected, surface‐sterilised and homogenised. The homogenates were cultured on NA plates to confirm the presence or absence of *E. hormaechei* .

## 3 Results

### 3.1 Molecular Analysis

Sequence alignment of the *16S rDNA* and *gapA* genes was performed using the BLASTn tool on the NCBI nucleotide database. The sequences showed the highest similarity (above 98%) to those of *Enterobacter hormaechei* . The isolates 1f, 1k and 5i were submitted to the GenBank database and were included in the phylogenetic analysis alongside reference sequences obtained from published studies. To construct the phylogenetic trees, sequences of the *gapA* and *16S rDNA* genes from 20 related *Enterobacter* species were used. To accurately determine the phylogenetic position of the isolates, analyses were conducted using the *16S rDNA* and the housekeeping gene *gapA*, both separately and in combination. Analysis of the *16S rDNA* sequence revealed a similarity of over 99% with members of the *Enterobacter cloacae* complex, including *E. cloacae* and *E. hormaechei* . However, due to the high conservation of the *16S rDNA* gene among closely related species, its discriminatory power at the species level is limited (Figure 1). In contrast, the phylogenetic tree based on the *gapA* gene, which has higher resolution, clustered the isolates closely with validated strains of *Enterobacter hormaechei* subsp. *xiangfangensis*. This grouping was strongly supported by high bootstrap values (> 95), indicating a close evolutionary relationship (Figure 2). Furthermore, the combined phylogenetic analysis of both genes (*16S rDNA* and *gapA*) confirmed the previous findings, placing the isolates firmly within the *E. hormaechei* subsp. *xiangfangensis* clade. This analysis clearly distinguished the isolates from other members of the *E. cloacae* complex, such as *E. cloacae* sensu stricto, *E. asburiae* , *E. bugandensis*, *E. ludwigii* and *E. roggenkampii* (Figure 3). These results demonstrate that combining housekeeping genes such as *gapA* with *16S rDNA* provides a powerful approach for precise intraspecific identification of members within the *Enterobacter cloacae* complex. Accurate species‐level identification is crucial for understanding the epidemiology and clinical relevance of opportunistic and nosocomial pathogens.

These findings indicate that the use of complementary genes such as *gapA* alongside *16S rDNA* provides an effective tool for accurate intraspecific identification of bacteria within the *Enterobacter cloacae* complex. Precise species‐level identification is particularly important for opportunistic and clinically relevant bacteria, as it plays a critical role in epidemiological studies and infection control strategies.

The antibiotic susceptibility assay revealed that all three antibiotics inhibited the growth of *E. hormaechei* compared to the control. The mean diameters of the inhibition zones were as follows:

- 83% relative inhibition for streptomycin (largest inhibition zone),
- 53% for gentamicin and
- 37% for tetracycline.

**FIGURE 4:**
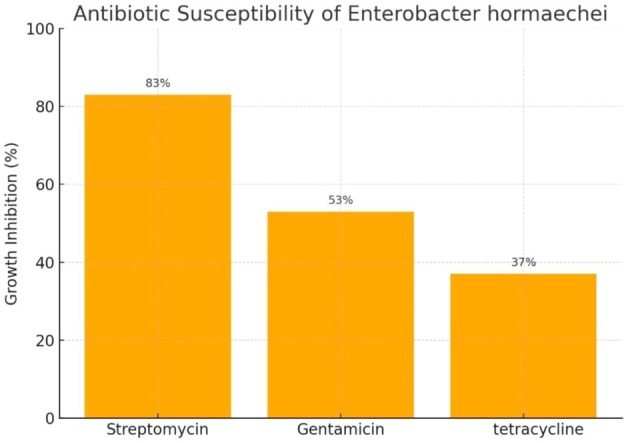
Percentage of *Enterobacter hormaechei* growth inhibition by different antibiotics.

**FIGURE 5:**
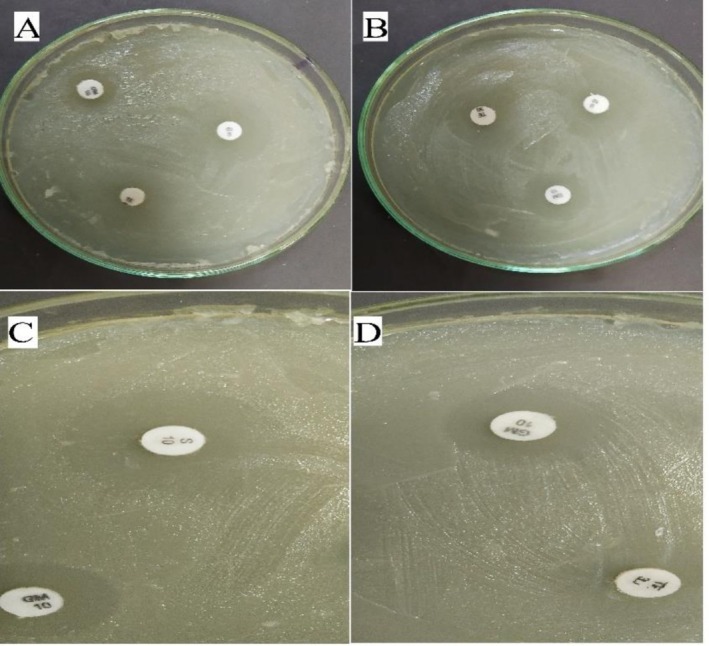
Inhibition of *Enterobacter hormaechei* growth by antibiotics on Nutrient Agar (NA) medium. (A and B) Inhibitory effect of three antibiotics—streptomycin (S), gentamicin (GM) and tetracycline (TE)—on bacterial growth. (C) Growth inhibition by streptomycin (S) and gentamicin (GM). (D) Growth inhibition by tetracycline (TE) and gentamicin (GM).

Based on these results, streptomycin was selected as the most effective antibiotic for the feeding assay (Figures 4 and 5).

**FIGURE 6:**
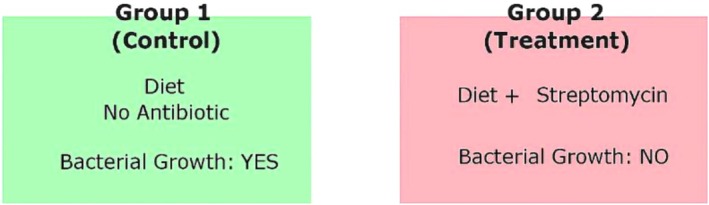
Schematic representation of the main experiment. Mites were divided into two groups: Group 1 (Control) received a diet without antibiotics and showed bacterial growth, while Group 2 (Treatment) received a diet containing streptomycin, which resulted in no bacterial growth.

**FIGURE 7:**
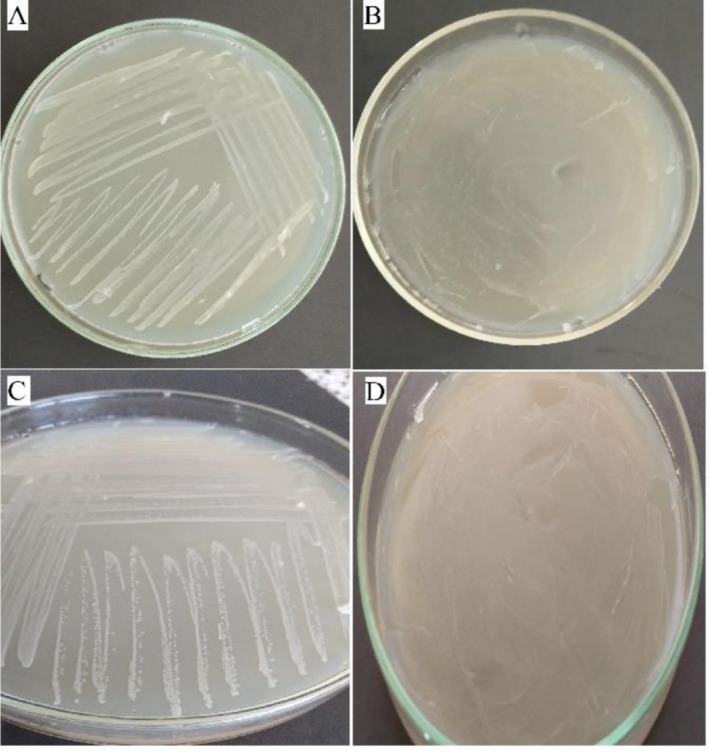
Effect of the antibiotic streptomycin on the growth of symbiotic bacteria isolated from mites. (A, C) Bacterial colonies isolated from mites fed on a diet without antibiotics (top and side views, respectively) show visible growth. (B, D) No bacterial growth observed from mites fed on a diet containing streptomycin (top and side views, respectively).

In the feeding experiment, viable and stable colonies of *E. hormaechei* were observed in the control group (mites fed with an antibiotic‐free diet), confirming the natural presence of the bacterium in the mite. In contrast, no bacterial growth was detected in the treatment group (mites fed with a streptomycin‐containing diet). The complete elimination of *E. hormaechei* in the treatment group, compared with its stable presence in the control, strongly indicates a natural and persistent symbiotic relationship between the bacterium and *C. lactis* (Figures 6 and 7).

## 4 Discussion and Conclusion

*Carpoglyphus lactis* is an environmental mite species that thrives in mouldy stored food products and has been implicated in allergic conditions such as allergic rhinitis, asthma and dermatitis (Arlian 2002). Heavy infestations of this mite in humid storage environments can exacerbate respiratory sensitivities (Fain et al. 1990). Although there is currently no direct evidence indicating *C. lactis* as a vector for *Enterobacter hormaechei* , environmental mites in general have the potential to act as carriers of opportunistic bacteria (Hubert et al. 2021). Microbiome studies of mites have shown that *Enterobacter* species, including *E. cloacae* and *E. hormaechei* , are stably associated endosymbionts that contribute to vital biological processes such as digestion, vitamin synthesis and modulation of the host immune system (Erban and Hubert 2011; Husník et al. 2011). This symbiotic relationship can provide the host mite with increased resistance to environmental stressors and protection against pathogenic microbes. Moreover, mites—especially astigmatid storage mites—are increasingly being recognised as potential reservoirs or vectors of antibiotic‐resistant bacteria, raising concerns regarding the zoonotic transmission of resistant genes (Brites‐Neto et al. 2015). The presence of resistant *Enterobacter* strains in mites may enhance the risk of gene transfer to humans or companion animals, particularly in agricultural or domestic settings where close contact is more likely. Symbiosis with *Enterobacter* may confer several advantages to the mite host, such as improved stress tolerance, nutritional efficiency and protection against pathogens. These interactions may influence mite biology and ecological success, and warrant further investigation into the role of the microbiome in mite health and behaviour.

Given the clinical and ecological significance of *Enterobacter* species, a detailed exploration of their interactions with mites can enhance our understanding of microbiome‐mediated survival, distribution and pathogenic potential of mites. Such insights could support the development of new strategies for managing mite populations and preventing the spread of microbial threats in both public health and agricultural contexts. *Enterobacter* *hormaechei* is one of the most important members of the *Enterobacteriaceae* family (Mezzatesta et al. 2012; Davin‐Regli and Pagès 2015). Accurate identification of this bacterium is essential for effective treatment of infections. It is a Gram‐negative, rod‐shaped bacterium that appears red in Gram staining due to its cell wall being rich in lipopolysaccharides (Sanders Jr and Sanders 1997). Biochemically, *E. hormaechei* is oxidase‐negative and catalase‐positive (Brady et al. 2013). Regarding carbohydrate metabolism, it ferments glucose and mannose without gas production and is also capable of fermenting trehalose (Stiles and Ng 1981; Mezzatesta et al. 2012). These characteristics can be useful in differentiating this bacterium from other species. The ability to grow in media containing 5% NaCl allows *E. hormaechei* to thrive under high osmotic stress conditions, such as those found in hospital environments (Davin‐Regli and Pagès 2015). Additionally, *E. cloacae* typically does not produce pigment on King's B agar, distinguishing it from pigment‐producing bacteria such as *Pseudomonas aeruginosa* (King et al. 1954). Overall, the biochemical features of *E. hormaechei* , including sugar fermentation profiles, salt tolerance and enzymatic activities, serve as key tools for its identification and differentiation from other species, playing a critical role in the diagnosis and treatment of infections caused by this pathogen. As a result, based on biochemical tests, it was determined that strains 1f, 1k and 5i are similar to the type strain ATCC 49162 of *Enterobacter hormaechei* (O’hara et al. 1989) (Table 1).

**TABLE 1:** Results of biochemical tests of three strains of *E. hormaechei* .

Biochemical test	*E. hormaechei* strain 1f, 1k and 5i	*E. hormaechei* type strain ATCC49162
Gram stain	Negative (−)	Negative (−)
Catalase test	Positive (+)	Positive (+)
Oxidase test	Negative (−)	Negative (−)
Fluorescent pigment on KB medium	Negative (−)	Negative (−)
Gas production from glucose	Positive (+)	Positive (+)
Oxidative/fermentative test	Fermentative (F)	Fermentative (F)
Growth in 5% NaCl	Positive (+)	Positive (+)
Acid Production from trehalose	Positive (+)	Positive (+)
Mannose utilisation	Usually positive (+)	Usually positive (+)

**TABLE 2:** Primer sequences used in this study.

Primer sequence (5′ → 3′)	Tm (°C)	Length (bp)	Primer name
GTCCGGAAAGAAATCGCTT	58	19	27F—16S rDNA
GCGGGACTTAACCCAACATC	58	20	1492R—16S rDNA
ATCTTCCTGACCGACGAAACTGC	61	23	gapA326F
ACGTCATCTTCGGTGTAACCCAG	61	23	gapA845R

The findings of this study demonstrate that *Enterobacter hormaechei* acts as a natural endosymbiont of the mite *C. lactis* . The antibiotic susceptibility assay, based on inhibition zone measurements, confirmed that streptomycin exhibited the greatest inhibitory effect on bacterial growth. The large inhibition zone surrounding streptomycin disks indicated its strong antibacterial activity, consistent with its known mechanism of action through interference with ribosomal protein synthesis (Jayarama 1962). The feeding assay provided further evidence for the symbiotic role of *E. hormaechei* . While mites fed with antibiotic‐free diets retained stable bacterial colonies, no bacterial growth was observed in mites exposed to streptomycin. The complete elimination of *E. hormaechei* in the treatment group clearly demonstrates that this bacterium maintains a stable and persistent presence within *C. lactis* . Previous studies have similarly reported that removal of symbiotic bacteria in ticks and insects using antibiotics can result in physiological changes, reduced reproduction and impaired digestion (Kolo and Raghavan 2023; Hussain et al. 2022). Thus, the present study not only confirms the endosymbiotic relationship between *E. hormaechei* and *C. lactis* but also underscores the ecological and physiological importance of bacterial symbionts in mite biology.

**FIGURE 8:**
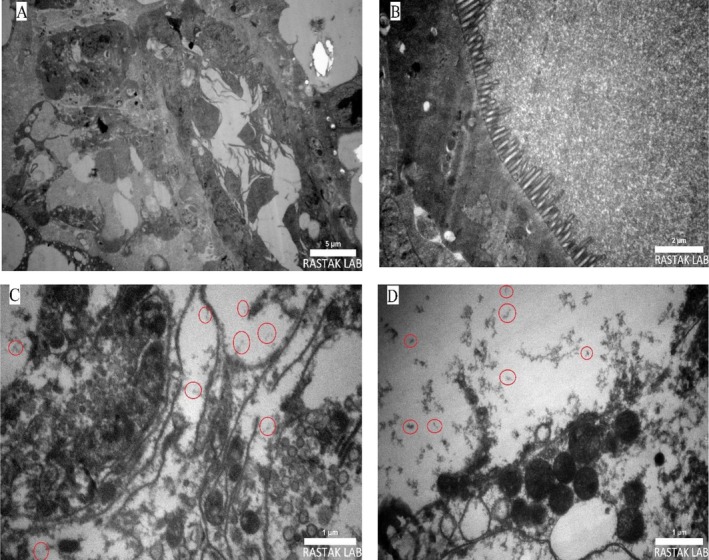
Transmission electron microscopy (TEM) micrograph of the mite *Carpoglyphus lactis*; (A) midgut and hindgut region of the mite (5 μm), (B) intestinal section (2 μm), (C) Bacillus‐shaped bacteria observed within the midgut (1 μm), and (D) Bacillus‐shaped bacteria observed within the hindgut (1 μm).

Transmission electron microscopy (TEM) further confirmed the presence of bacteria within the midgut and hindgut of *C. lactis* . The micrographs revealed bacterial structures aggregated within the epithelial cells of the digestive tract. One of these bacteria was identified as *Enterobacter hormaechei* , which was isolated and characterised using molecular and biochemical methods. These findings provide direct evidence of a stable endosymbiotic relationship within the mite's gut tissues, emphasising the crucial role of the microbiome in the physiology and environmental adaptation of this species (Figure 8).

## Author Contributions

**Majid Rakhshandeh:** investigation, writing – original draft, writing – review and editing, validation, methodology, software, formal analysis, data curation, resources. **Mohammad Khanjani:** project administration, funding acquisition, writing – review and editing, supervision, resources, formal analysis, validation.

## Conflicts of Interest

The authors declare no conflicts of interest.

## Data Availability

The data that support the findings of this study are available on request from the corresponding author. The data are not publicly available due to privacy or ethical restrictions.
